# Electroencephalographic Abnormalites in SARS-CoV-2 Patients

**DOI:** 10.3389/fneur.2020.582794

**Published:** 2020-11-26

**Authors:** Stephane Besnard, Clotilde Nardin, Elsa Lyon, Thomas Debroucker, Roxana Arjmand, Raffaella Moretti, Hervé Pochat

**Affiliations:** ^1^University Hospital of Caen Normandie, Explorations Fonctionnelles, CHU Caen, Caen, France; ^2^Centre Hospitalier Delafontaine, Saint-Denis, France; ^3^ELYOPE SAS, Paris, France; ^4^Centre Hospitalier de Sens, Yonne, Sens, France; ^5^Explorations Fonctionnelles Pédiatriques, CHU Armand-Trousseau, APHP, Paris, France; ^6^SIGMA EEG SARL, Paris, France

**Keywords:** confusion, epileptic seizure, virus, encephalopathy, electroencephalography

## Abstract

Viral infection with SARS-CoV-2 has a neurological tropism that may induce an encephalopathy. In this context, electroencephalographic exploration (EEG) is indicated as a diagnostic argument correlated with lumbar puncture, biology, and imaging. We performed a retrospective analysis of 42 patients explored by EEG and infected by COVID-19, according to the EEG abnormalities and clinical signs that motivated the examination. Confusion and epileptic seizures were the most common clinical indications, with 64% of the patients displaying these symptoms. The EEG was altered in 85% of the cases of confusion, in 57% of the cases of epileptic symptoms (general or focal seizure or prolonged loss of contact) and 20% of the cases of malaise or brief loss of consciousness. Nine EEG (21%) were in favor of an encephalopathy, two had *de novo* alterations in persistent consciousness and two had alterations in general states of confusion; one was very agitated and without history of epilepsy and combined eyelids clonia while a second one exhibited unconsciousness with left hemicorpus clonus. Two were being investigated for delayed awakening without sedation for more than 24 h. All of these patients were diagnosed COVID-19, some of them with associated mild to severe respiratory disorders. This work shows the interest of the EEG in exploring COVID-19 patients suffering from neurological or general symptoms looking for cerebral alteration.

## Introduction

The current pandemic viral infection with coronavirus 2 (SARS-CoV-2) appears to have, as its initial target, the respiratory tract inducing acute respiratory distress syndrome, particularly in elderly subjects with certain risk factors including diabetes, immunosuppression, and chronic renal and respiratory failure. As with any severe viral infection, there is a risk of dissemination to the central nervous system with general neurological symptoms such as fatigue, headache, confusion, myalgia, and more specifically anosmia and agueusia (1). Neurological impairments may result in an encephalopathy, meningoencephalitis, necrotizing encephalitis (2) documented by imaging (3) and lumbar puncture (4) and may be accompanied by epileptic seizures or stroke (5, 6). This neurological impairment seems to be correlated with the severity of the infection (7). The underlying neurophysiopathological mechanism remains to be clarified and appears to be multimodal. The virus could cross the blood-brain barrier and bind to hACE2 receptors co-expressed with acetylcholine receptors; could induce immunological reaction; and could penetrate through the olfactory mucosa and then the receptors and olfactory nerves as entry points (1, 8, 9). Moreover, the neurological damages could be due to or aggravated by cerebral hypoxemia and metabolic acidosis induced by respiratory disorders (10, 11). Respiratory disorders could in turn be aggravated by a dysfunction of the respiratory centers, located in the brainstem, a predominant target of SARS-CoV-2 as demonstrated in a mouse model of infection (10). In total, cerebral impairments could express or combine three encephalopathic types: an infectious toxic encephalopathy, a viral encephalitis, and an anoxic encephalopathy, as described by Wu et al. (12).

The electroencephalogram (EEG), which is one of the tools for neurological explorations, could be of interest in the diagnosis of encephalopathy in the context of patients with COVID-19 and those suffering from neurological symptoms. Indeed, a clinical case has reported a man of 74 years-old suffering from respiratory distress associated with mental confusion who presented EEG abnormalities in the form of diffuse slowing and focal slowing sharply contoured waves in the left temporal region. However, while the diffuse abnormalities could be related to encephalopathy, the focal abnormalities appeared to be related to encephalomalacia secondary to a previous stroke (5).

In addition, pre-existing neurological pathology, particularly epilepsy, could be aggravated by a SARS-CoV-2 infection according to its neurological tropism. Consequently, we retrospectively analyzed and reported the EEG patterns of 42 patients infected by SARS-CoV-2.

## Methods

This is a retrospective study from an EEG database interpreted by neurophysiologists with the help of the SIGMA EEG Company, supporting facilities and administrative procedures for the transfer of medical exams whose EEG. Clinical information was collected from information sent by prescribers from Center Hospitalier Delafontaine, Center Hospitalier de Sens, Center Hospitalier Sainte Camille, Center Hospitalier de Joigny, Center Hospitalier de Brie Comte Robert, and Center Hospitalier de Coulomiers. Two EEGs were included and classified according to the indication of the exam and the electrophysiological abnormalities observed. Indications were classified as followed: (a) confusion or psychomotor retardation; b/clinical epileptic symptoms with generalized seizure or focal seizure, prolonged loss of consciousness with general hypotonia, (b) short loss of consciousness, (c) delayed awakening after reanimation, (d) hallucinations or behavioral disorders, (e) transitory ischemic stroke or suspected stroke; (f) follow-up of a meningoencephalitis. We have classified EEG abnormalities as follows: normal with somnolence, slight slowdown rhythm or poorly organized, some non-specific abnormalities, focal or diffuse epileptic pattern, encephalopathic pattern. The results are presented in a descriptive manner like a case report.

## Results

Patients included were referred for EEG over a 2-month period between March and April 2020. Twelve EEGs were normal (21.8%), 9 showed a slight deceleration without spatial organization (21.4%), 8 some non-specific abnormalities or questionable elements (19%), 4 focal or diffuse epileptic EEG abnormalities including one related to symptomatic focal epilepsy related to stroke prior to SARS-CoV-2 infection (9.5%). Nine showed an encephalopathic pattern, one of the patients being still under sedation (21.4%) (Table 1).

**Table 1:** Distribution of the electroencephalographic patterns observed in patients infected by CoV-SAR-2.

**EEG pattern in COVID 19 + patients**	**Absolute values**	**%**
Normal with drowsiness	12	28.6
Slight slowdown rhythm or poor spatial organization	9	21.4
Unspecific anomalies	8	19
Focal or diffuse epileptic pattern (diffuse spike and polyspikes, frontal spikes, temporal, and rolandic slow sharp waves or spikes and wave spikes, and altered sharp waves	4	9.5
Encephalopathic pattern (continuous or rhythmic frontal or diffuse slow diphasic or triphasic waves or sharp waves)	9	21.4
Total	42	

On 33% of patients with confusion or psychomotor alterations, two EEG were normal with drowsiness, 3 EEGs were slowed but one under midazolam, 4 had some abnormal non-specific features, two had epileptic anomalies (one with rolandic epileptic abnormalities or lateralized epileptiform discharges at 1 Hz (LPDs) probably more related to a previous stroke, not fullfing criteria for non-convulsive status epilepticus; and one with a status epilepticus (fronto-temporal slow waves spikes at 2 Hz) solved with intravenous clonazepam injection) and 3 had an encephalopathic pattern. The EEG was therefore modified in 85% of the cases of confusion.

Out of 30.9% of patients with clinical epileptic symptoms, 5 EEG were normal, 1 was slowdown, 2 presented non-specific abnormalities, 2 were with comital abnormalities (one with frontal sharp-waves epileptic seizures and one with focal rolandic sharp-waves and spikes with or without slow waves) and three in favor of an encephalopathy but one remained under sedation. The EEG was then altered in 57% of cases.

Patients with epileptic symptoms expressed general tonico-clonic seizures or focal clonic seizure (limbs or jaw).

Of 12% of patients with brief loss of consciousness, three had normal EEGs, one was slightly slowdown and one showed encephalopathic pattern, so we had 20% EEG changes in case of brief loss of consciousness.

Regarding the EEG traces on hallucination, one was normal but raised doubts about pharmacological rhythms and the second one was unspecifically slowed down. The EEG for suspicion of transitory ischemic crebrovascular impairment was normal. Of the EEGs for delayed awakening, two were slightly slowed down in rhythm, three showed unspecific abnormalities, and one had a pattern of encephalopathy (Table 2).

**Table 2:** Percentage of abnormal EEGs according to their pattern and the initial indication of EEG.

**Indication of the EEG and %**		**EEG reports (number of patients)**				
	**% from (the number of patients)**	**Normal with drowsiness**	**Slight slowdown, poor spatial organization**	**Unspecific anomalies**	**Focal/diffuse comitial anomalies, PLEDS or similar**	**Encephalopathic pattern**
Confusion/psychomotor retardation	33.3 (14)	2	3	4	2	3
Epileptic seizure (general tonico-clonic or focal clonic seizure, prolonged loss of consciousness with general hypotonia)	30.9 (13)	4	1	2	2	3
Short loss of consciousness	11.9 (5)	3	1	0	0	1
Delayed awakening after reanimation	14.3 (6)	0	2	2	0	2
Hallucinations/altered behavior	4.7 (2)	1	1	0	0	0
Transitory ischemic disease	2.4 (1)	0	1	0	0	0
Follow-up of a meningoencephalitis	2.4 (1)	1	0	0	0	0
Total	42	12	9	8	4	9

**Figure 1:**
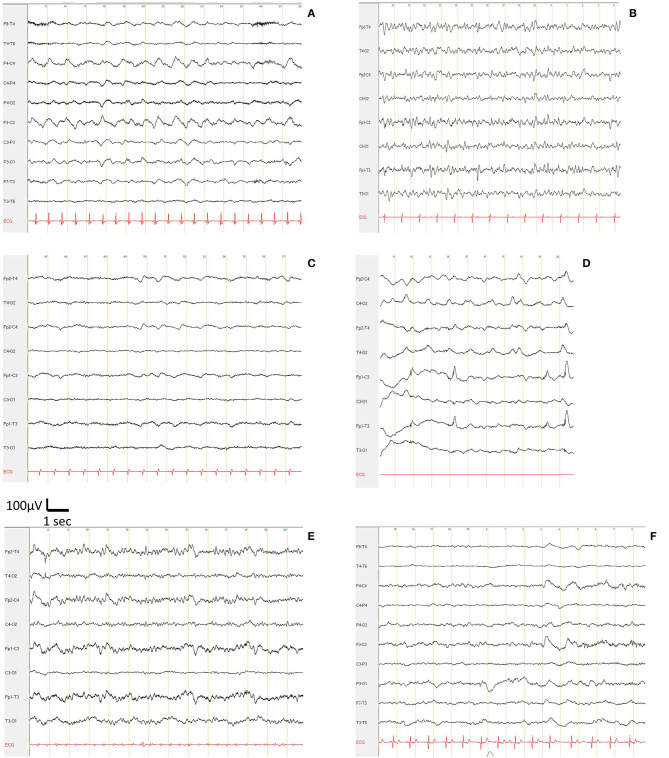
Encephalopathic pattern with triphasic frontal waves and a frequency of the basal rhythm from 1 to 7 Hz, in COVID patients suffering from suspicion of a status epilepticus **(A)**, syncope **(B)**, delayed awakening after reanimation and being weaned off anesthetic drugs **(C)**, bilateral eyelid myoclonus during awakening following reanimation (**D**, artifacts on Fp1), altered consciousness **(E)**, and confusion for 2 days **(F)**.

Of 21.4% with an EEG in favor of encephalopathy (Figure 1), two had alterations without disorders of consciousness and two had alterations in general state with confusion; one was very agitated and without history of epilepsy and combined palpebral clonia after sedation had been stopped for more than 24 h while a second one exhibited unconsciouness with left clonies. Two were being investigated for delayed awakening without sedation for more than 24 h, one was being investigated for unconsciousness but his clinical condition at the time of examination had deteriorated rapidly with disturbances of consciousness, and one was being investigated for a suspicion of a state of illness in a known epileptic patient who had received anti-epileptic treatment and sedation. All of these patients were diagnosed COVID+, some of them with associated mild to severe respiratory disorders. For patients being investigated for delayed awakening, the clinical state was obviously severe as they required intensive care. Meningeal or cerebral damage remains difficult to prove and not all paraclinical elements were available at the time of the EEG.

The respective role of epilepsy and COVID-19 in neurological involvement remains subject of caution. Indeed, any infection may aggravate an existing epilepsy through hyperthermia, inflammatory syndrome, or cerebral tropism, although epileptic symptoms, with no known history of epilepsy, could be an initial expression of neurological damage.

## Discussion

EEG in a patient who is suspect or positive for COVID 19 was mainly prescribed from signs of encephalopathy or seizure as previously reported (13–15). Out of 42 EEGs performed, 9 were suggestive of encephalopathy. This encephalopathic aspect may be linked to viral involvement but should be discussed according to the level of sedation during the examination and also to suffering related to hypoxemia. One study reports nearly 41% of epileptiform abnormalities with 88% of frontals sharp waves. The proportion of EEG anomalies in favor of encephalopathy was 18% (14) for 21% in our study while Pellinen et al. (15) reported moderated generalized slowing for 57%. In addition, a previous spectral analysis study of the EEG confirmed the electrical changes in case of encephalopathy, even suggesting the ability to differentiate between infectious toxic encephalopathy on the one hand, and from encephalopathies in a context of severe hypoxia on the second hand (16). The proportion of altered EEG of about 85% reported here regardless the medical indication, was similar to those previously reported (15, 17). Viral infection with COVID 19 in patients with epilepsy may trigger or worse epileptic seizures more easily, particularly in the case of genetic abnormalities (18).

The EEG performed in the context of exploring delayed awakening remains difficult to date and impossible to correlate with specific central neurological damage related to COVID 19. Indeed, residual sedation, initial hypoxemic suffering and neurological damage may combine and induce EEG abnormalities of different kinds that can be assimilated to an aspect of encephalopathy.

The electroencephalographic observations confirm neurological impairment in the context of SARS-COV-2 infection, as previously shown by postmortem analysis for the mesencephalon (hypothalamus) and the cortex (19). However, electrical abnormalities on the EEG remain non-specific and cannot make the diagnosis of neurological impairment by SARS-COV-2 as previously reported (17, 20).

It remains difficult to correlate EEG abnormalities with cerebral MRI, lumbar puncture and thoracic CT since cerebral MRI and lumbar puncture were not routinely performed and the entire medical record could not be reported on the telemedicine platform on which the EEGs were interpreted. In a subgroup of 13 patients, we were able to obtain the results of the thoracic CT scan, PCR and lumbar puncture. We had no correlations between these items (data not shown). Moreover, the timing of EEG in the timeframe of the medical investigations remains difficult to collect as it was performed according to the onset of the neurological symptoms and not pulmonary or other first symptoms.

The pathophysiological mechanism(s) probably remain multimodal: viral encephalitis, infectious toxic encephalopathy or cerebrovascular involvement as proposed by Wu et al. (12). The encephalopathic aspect of the EEG was reported in a case report in a 74-year-old patient suffering from a SARS-COV-2 viral infection with pulmonary and neurological damage. The EEG showed electrical signs of encephalopathy and a slow temporal focus that was more likely to be related to a history of left temporal stroke with leukomalacia on imaging (5).

Damage to the olfactory nerve, thalamus and brain stem was demonstrated in a mouse model with intranasal injection of the virus (10). The brain stem appears to be the most affected site (10). Therefore, it might be relevant to systematically explore, in the case of neurological impairment, to add auditory, visual, and somatosensory evoked potentials in the assessment.

Confusion and seizure were the main indicators associated with an EEG aspect of encephalopathy. It is suspected that SARS-COV-2 infection may aggravate seizures in a patient with a history of epilepsy or being monitored for epilepsy. However, of the 13 patients with seizures, only three had a history of epilepsy and for two patients we did not have the information.

The EEG was performed at the time of onset of neurological clinical signs, but the delay between the EEG and the onset of respiratory clinical signs, for patients who had suffered from these, remains difficult to quantify. For some patients, the EEG was performed in the first few days, for others 15 days later and finally for patients with delayed recovery after resuscitation for up to 3 weeks. There was also no correlation between the degree of chest CT and encephalopathic pattern on the EEG on a subgroup of 13 patients for whom we had the imaging report. For those patients who ultimately had a negative PCR reported to us afterwards, the EEG remained normal.

Finally, given the percentage of abnormalities regardless of the initial indication, the EEG remains a useful test to explore any patient infected with COVID 19 with neurological signs.

EEG exploration after sedation remains difficult because of the pharmacological influence to discriminate the neurological damage linked to the COVID but seems interesting in some cases as previously reported (21).

## Data Availability Statement

The raw data supporting the conclusions of this article will be made available by the authors, without undue reservation.

## Ethics Statement

Ethical review and approval was not required for the study on human participants in accordance with the local legislation and institutional requirements. Written informed consent for participation was not required for this study in accordance with the national legislation and the institutional requirements.

## Author Contributions

SB has analyzed the EEG, data, and wrote the paper. CN, TD, RA, and RM have analyzed the EEG. EL and HP have analyzed the data and wrote the paper. All authors contributed to the article and approved the submitted version.

## Conflict of Interest

SB is consultant as expert for SIGMA EEG. EL and HP were employed by company ELYOPE SAS and SigmaEEG respectively. The remaining authors declare that the research was conducted in the absence of any commercial or financial relationships that could be construed as a potential conflict of interest.
